# Supplementary material for: UV-LED lights enhance the establishment and biological control efficacy of Nesidiocoris tenuis (Reuter) (Hemiptera: Miridae)
Source: PLoS One. 2021 Jan 7;16(1):e0245165. doi: 10.1371/journal.pone.0245165 (PMC7790406; doi:10.1371/journal.pone.0245165)
Supplement: Attachment — Submitted filename: Response to Reviewers.docx [file pone.0245165.s002.docx]

**Dear Editor**

Thank you for giving us another chance and the helpful comments on our manuscript. We have faithfully revised our manuscript by referring to the reviewer's opinion. And, according to the reviewer’s suggestion, our manuscript was professionally re-edited for English by "VanDriesche Scientific Editing". The address of Young-gyun Park was changed, thus we added: “Present address” on our title page. Also, we newly upload our figures with better resolution (Fig 8 was revised according to the reviewer’s comment). Moreover, according to the opinion of Reviewer 3, we changed the title to "UV-LED Lights Enhance the Establishment and Biological Control Efficacy of *Nesidiocoris tenuis* (Reuter) (Hemiptera: Miridae) in the Greenhouse".

Revised parts for reviewer 1, 2, and 3 were marked in red, green, and orange color in the text, respectively.

**Reviewer’s comments and our responses (Reviewer #1)**

**Major comments**

The manuscript intitled “Increase of control efficacy of *Nesidiocoris tenuis* (Hemiptera: Miridae) in the greenhouse by enhancing its establishment using UV-LED light” presents new finds concerning *B. tabaci* control by using *N. tenuis* and several kind of wavelengths. The introduction section should be enhanced, specifically concerning the biological traits of *B. tabaci* and *N. tenuis*. The objective of this work need be better explained. The manuscript presents several parts without link (i.e. i. why the authors used less insects in the experiment 2? ii. Why it was chosen the predator wavelengths preference in greenhouse experiment? Etc). Additionally, the manuscript is poorly referenced. Finally, the whole manuscript ought to be revised by a native speaker.

**Our response:** Thank you for the comments. We sincerely appreciate your suggestions. We revised our manuscript by referring to your suggestion. Your questions in major comments were answered in the minor comments section. And, according to the reviewer’s suggestion, our manuscript was professionally re-edited for English by "VanDriesche Scientific Editing"

**Minor comments
1. Reviewer comment:** L16: Replace “is a biological control agent for controlling” to “ is one of the biological control agents against..”

**Our response:** We changed it according to the suggestion. Thank you. Please check L18-19 in the revised manuscript.

**2. Reviewer comment:** L17: I suggest to use: “The successful..”, “pests..”, and “for effective..”

**Our response:** We changed it according to the suggestion and English editing process. Thank you. Please check L20-22 in the revised manuscript.

*Original sentence:* Successful establishment of a biological control agent and its spatial coherence with pest in the target area are essential for an effective biological control in greenhouses.

*Revised sentence:* The successful establishment of a biological control agent and co-occurrence with the target pests increases the efficacy of biological control in greenhouses.

**3. Reviewer comment:** L19-21: Why the authors want to attract *B. tabaci* for crops? In fact, you should attract natural enemies and repel pests. You need to write the goals more clearly.

**Our response:** Thank you for the nice question. However, unlike pheromone that can attract specific species, it is very tough to induce attraction response to one species using a certain wavelength of light with high efficiency. There were many reports about the positive response for UV or near-UV for pests and natural enemies. Some wavelengths of those ranges were finally chosen for enhancing the establishment of natural enemies [1-3]. In these results, applying a certain wavelength to attract specific species of insects might be hard. Thus, it would be better to consider the spatial congruence of both pests and natural enemies for enhancing the efficacy of biological control.

The statement in the abstract section in the original manuscript may cause some confusion, as the reviewer raised. Thus, we revised that sentence. Please check L22-24 in the revised manuscript.

*Original sentence:* In this study, we explored proper wavelengths of LED light that could attract both *B. tabaci* and *N. tenuis* and enhance their spatial coherence so that biological control of *B. tabaci* could be more effective.

*Revised sentence:* In this study, we explored the effects of different wavelengths of LED light on establishment of *N. tenuis,* with the goal of enhancing the biological control of *B. tabaci* in greenhouse crop.

References

1. Johansen NS, Vänninen I, Pinto DM, Nissinen AI, Shipp L. In the light of new greenhouse technologies: 2. Direct effects of artificial lighting on arthropods and integrated pest management in greenhouse crops. Ann Appl Biol. 2011; 159(1): 1-27.
2. Ogino T, Uehara T, Muraji M, Yamaguchi T, Ichihashi T, Suzuki T, et al. Violet LED light enhances the recruitment of a thrip predator in open fields. Sci Rep. 2016; 6: 32302.
3. Uehara T, Ogino T, Nakano A, Tezuka T, Yamaguchi T, Kainoh Y, et al. Violet light is the most effective wavelength for recruiting the predatory bug *Nesidiocoris tenuis*. BioControl. 2019; 64(2): 139-147.

**4. Reviewer comment:** L21: “LED light at a wavelength..”

**Our response:** We changed it according to the suggestion. Thank you. Please check L24-25 in the revised manuscript.

**5. Reviewer comment:** L21-22: Incomplete statement, revise.

**Our response:** We changed it. Thank you. Please check L24-25 in revised manuscript.

*Original sentence*: LED light at a wavelength of 385 nm attracted *N. tenuis* the most.

*Revised sentence*: *Nesidiocoris tenuis* was most strongly attracted by LED light at a wavelength of 385 nm.

**6. Reviewer comment:** L27: “at a wavelength..”

**Our response:** We changed it according to the suggestion. Also, we found same error in L25, and it was corrected. Please check L29 and 32 in the revised manuscript.

**7. Reviewer comment:** L32: *B. tabaci* is a polyphagous pest, as you showed in your text; it appears that *B. tabaci* is a problem only for the cultures cited. Revise!

**Our response:** We changed “greenhouse” to “horticultural” and added “etc.” after “flower”. Please check L38 in the revised manuscript.

**8. Reviewer comment:** L38-39: Explain better about alternatives to control *B. tabaci*.
References suggested:

1. FEKRI, Masoomeh Samareh et al. The combined effect of some plant extracts and pesticide Pymetrozine and two tomato varieties on biological characteristics of *Bemisia tabaci* (Homoptera: Aleyrodidae) in greenhouse conditions. Entomologia Generalis, p. 229-242, 2016.
2. SOARES, Marianne A. et al. Detrimental sublethal effects hamper the effective use of natural and chemical pesticides in combination with a key natural enemy of *Bemisia tabaci* on tomato. Pest Management Science, 2020.
3. DONG, Yong‐Cheng et al. Nitrogen and water inputs to tomato plant do not trigger bottom‐up effects on a leafminer parasitoid through host and non‐host exposures. Pest management science, v. 74, n. 3, p. 516-522, 2018.
4. LYKOURESSIS, Dionyssios P. et al. Predation rates of *Macrolophus pygmaeus* (Hemiptera: Miridae) on different densities of eggs and nymphal instars of the greenhouse whitefly *Trialeurodes vaporariorum* (Homoptera: Aleyrodidae). Entomologia generalis, v. 32, n. 2, p. 105-112, 2009.

**Our response:** Thank you for the brilliant suggestion. We changed it according to the suggestion with references. However, your suggestion's 4th reference was omitted in our revision because it was a study for Trialeurodes vaporariorum, not Bemisia tabaci. Please check L44-47, 557, 564, and 568 in the revised manuscript.

*Original sentence*: Thus, biological control has been increasingly promoted for the control of *B. tabaci*.

*Revised sentence*: Therefore, alternative control strategies such as combinations of pesticides and plant extracts, manipulations of tri-trophic effects, and biological control have become of increasing interest for control of *B. tabaci.*

**9. Reviewer comment:** L44-45: References suggested about the control of pests provided by *N. tenuis*.

1. BIONDI, Antonio et al. Can alternative host plant and prey affect phytophagy and biological control by the zoophytophagous mirid *Nesidiocoris tenuis*?. BioControl, v. 61, n. 1, p. 79-90, 2016.
2. CAMPOS, Mateus R. et al. From the Western Palaearctic region to beyond: *Tuta absoluta* 10 years after invading Europe. Journal of Pest Science, v. 90, n. 3, p. 787-796, 2017.
3. BIONDI, Antonio et al. Ecology, worldwide spread, and management of the invasive South American tomato pinworm, *Tuta absoluta*: past, present, and future. Annual Review of Entomology, v. 63, p. 239-258, 2018.
4. SOARES, Marianne A. et al. Botanical insecticide and natural enemies: a potential combination for pest management against *Tuta absoluta*. Journal of Pest Science, v. 92, n. 4, p. 1433-1443, 2019.
5. DESNEUX, Nicolas et al. Biological invasion of European tomato crops by *Tuta absoluta*: ecology, geographic expansion and prospects for biological control. Journal of pest science, v. 83, n. 3, p. 197-215, 2010.

**Our response:** Thank you for the brilliant suggestion. We added the suggested references in our manuscript. Please check L50-51, 586, 600, 603, 610, and 613 in the revised manuscript.

**10. Reviewer comment:** L53: Replace “settlement” to “establishment”

**Our response:** We changed it according to the suggestion. Please check L63 in the revised manuscript.

**11. Reviewer comment:** L70: As I told before, the objectives should be clearer.

**Our response:** Thank you for the suggestion. We revised our sentences. Please check L86-95 in the revised manuscript.

*Original sentences*: If a selected LED light shows asymmetrical response between *N. tenuis* and its prey, it could result in spatial separation between the predator and the prey. Therefore, the objective of this study was to explore the proper wavelength of LED light to attract both *B. tabaci* and its predator, *N. tenuis*, and determine if enhancing spatial coherence of these two species using LED light could successfully control *B. tabaci* in a tomato greenhouse.

*Revised sentences*: If a selected LED light is asymmetrically attractive between *N. tenuis* and its prey, the spatial separation between the predator and the prey, rather than being reduced, might increase. Therefore, we hypothesized that the best strategy would be to select LED light wavelengths that have high attractiveness for both the predator and prey. The specific objectives of this study, therefore, were to determine if selected wavelengths of LED light would enhance establishment of *N. tenuis* and thus improve biocontrol of *B. tabaci* in a tomato greenhouse. Additionally, the responses of *B. tabaci* to selected wavelengths of LED light were explored to determine their impact on the spatial congruence of the distributions of natural enemies and the target pest.

**12. Reviewer comment:** L82: What does meaning “for ca. 1 h”? Unclear!

**Our response:** We used “ca.” as a mean of “about”. So, we changed it to “about”. Please check L102 in the revised manuscript.

**13. Reviewer comment:** Figure legend: Provide all legends in a single page. Consequently, you should remove these legends from the continuous text.

**Our response:** We followed the formatting guidelines of PLoS ONE (<https://journals.plos.org/plosone/s/file?id=wjVg/PLOSOne_formatting_sample_main_body.pdf%20>).

We followed the guideline below.

**14. Reviewer comment:** L84: Remove both “(S.D.)”

**Our response:** Remove done. Please check L105 in the revised manuscript.

**15. Reviewer comment:** L90: Replace “*B. tabaci*” to “*Bemisia tabaci*”. After dot all scientific names should write unabbreviated

**Our response:** We changed it. Please check L110 in the revised manuscript.

**16. Reviewer comment:** L94: Did you observe cannibalism between nymphs and adults?

**Our response:** We gave enough eggs of *C. cautella* as food for *N. tenuis*. We observed so many times when we changed the food or just wanted to observe, and the nymphs and adults always got along well.

**17. Reviewer comment:** L96: I totally disagree with all title of the experiments. In fact, the title is a brief of each experiment. Revise all!

**Our response:** Revision was done.

Test insects -> Insects sources and rearing

Y-tube experiments -> Exp. #1: Wavelength selection (in Y-tube tests)

Incubator experiment for verification of enhanced establishment rate

-> This part was divided into two chapters.

-> Exp. #2: No-choice test of effect of light treatment on *N. tenuis* movement and predator efficiency

-> Exp. #3: Choice test of effect of light treatment on *N. tenuis* movement and predator efficiency

Greenhouse experiment

-> This part was divided into two chapters.

-> Exp. #4: Effects of selected wavelengths on predator and prey in a greenhouse trial

-> Exp. #5: Second tomato greenhouse trial

Please check L98, 117, 146, 204, 214, 267, 296, 321, 350, and 400 in the revised manuscript.

**18. Reviewer comment:** L98: “To find the proper”

**Our response:** We revised the whole of the sentence with English editing. Please check L118-120 in the revised manuscript.

*Original sentence*: Y-tube (200 mm in length and 40 mm in diameter for each branch) tests were conducted to find proper wavelength of LED light that would shows the highest attraction rate for *N. tenuis* and *B. tabaci*.

*Revised sentence*: Wavelength selection experiments were conducted to find the wavelength of LED light that would attract both *N. tenuis* and *B. tabaci* by using a Y-tube assay (200 mm in length and 40 mm in diameter for each branch).

**19. Reviewer comment:** L110: Male or female? Age? Provide in the manuscript.

**Our response:** We concentrated on the response of the group; thus, we randomly collected from the colony. We added “randomly selected from the purchased bottle” for *N. tenuis* and “randomly selected from the rearing colony” for *B. tabaci*. Please check L135-136 and L138-139 in the revised manuscript.

**20. Reviewer comment:** L111: Did you use a yellow trap? Provide in the manuscript.

**Our response:** No, we used the transparent sticky traps for Y-tube test. Actually, we already mentioned it in the same section. However, we added “transparent” according to your suggestion. Please check L140 in the revised manuscript.

**21. Reviewer comment:** L113-115: Move to “statistical analysis” section

**Our response:** Move done. Please check L280-282 in the revised manuscript.

**22. Reviewer comment:** L122: Remove both “(S.D.)”

**Our response:** Remove done. Please check L156 in the revised manuscript.

**23. Reviewer comment:** L127: How many insects were used in each replication?

**Our response:** Ten *N. tenuis* were used for the first experiment (Exp. #2) in an incubator. And, 15 *N. tenuis* were used for the second experiment (Exp. #3) in an incubator. We mentioned this in our manuscript. Please check L186 and 211 in the revised manuscript.

**24. Reviewer comment:** L131: Replace “petri” to “Petri”. Revise the complete manuscript.

**Our response:** We changed all in our manuscript. Please check L165, 166, 171, 172, 187, 189, and 193 in the revised manuscript.

**25. Reviewer comment:** L135: Replace “to provide refuge” to “to provide refuge and food substrate”

**Our response:** Thank you. correction was done. Please check L169 in the revised manuscript.

**26. Reviewer comment:** L135: Replace “also” to “additionally”

**Our response:** We revised the whole of the sentence with English editing. Please check L169-173 in the revised manuscript.

*Original sentence*: Also, 100 eggs of *C. cautella* on a small Petri dish (40 mm x 6 mm; diameter x height) were provided as food in each large Petri dish (100 mm x 42 mm; diameter x height).

*Revised sentence*: To measure predation, feeding on a non-pest was used as a predation index to compare treatments. This index was the rate of predation on 100 eggs of *C. cautella*, which were placed on a small Petri dish (40 mm x 6 mm; diameter x height), which itself was placed on the large Petri dish (100 mm x 42 mm; diameter x height) on which the tomato stems had been placed.

**27. Reviewer comment:** L163-165: Move to “statistical analysis” section

**Our response:** Move done. Please check L283-286 in the revised manuscript.

**28. Reviewer comment:** L169-176: In my opinion, this part is useless. You can just mention that this preliminary test was carried out before the real experiment.

**Our response:** We deleted that part in M & M and the results. And, we added a relevant sentence in M & M of the incubator experiment (Exp. #2 and #3). And also, the figure legend was moved into that part and. In the figure legend, we added information about the test method. Please check L179-180, 199-202, 214 in the revised manuscript.

*Added sentence in M & M part of incubator experiment*: The timing of when the additional lighting occurrence was based on data from a preliminary test in a greenhouse (Fig 1).

*Figure legends*: Fig 1. Number (Mean ± S.E.) of *N. tenuis* adults attracted to 385 nm wavelength LED light (9-W) developed in a greenhouse (14.5 x 7 m^2^; width x length) without plants (three replications, 100 *N. tenuis* in each replication) at different points in the daily light cycle.

**29. Reviewer comment:** L200-202: You can omit this information.

**Our response:** Correction was done. Please check L243 in the revised manuscript.

**30. Reviewer comment:** L210: Why in the experiment 2 less insects than the experiment 1 were released?

**Our response:** Thank you for the question. As we mentioned in the discussion, the initial environmental conditions of the Exp. #4 (revised version of experiment 1) were unsuitable for both *B. tabaci* and *N. tenuis*. Actually, no heating system was operated, unlike real commercial cultural greenhouses. Densities of both insects in all treatment plots were consistently low until June 12. Thus, we released *N. tenuis* and *B. tabaci* several times. On the contrary, after transplanting of tomato plants to the greenhouse in the Exp. #5 (revised version of experiment 2), the external inflow of *B. tabaci* (5 to 10 per plant) occurred. Thus, we released less number of insects in the Exp. #5.

**31. Reviewer comment:** L218-227: You should provide this information in your table. Think to optimize how to show the information in your manuscript.

**Our response:** We added the content of L218-227 in Table 1, and revised that part. Please check L246 and 253-257 in revised manuscript.

*Original sentences*: First, 100 adults of *B. tabaci* were released to all plots on April 24 and 150 adults of *B. tabaci* were additionally released on April 30 before the first observation day. Then seven adults of *N. tenuis* were released to <*B. tabaci* + *N. tenuis*> and <*B. tabaci* + *N. tenuis* + LED> plots on May 9. The optimal release number of *N. tenuis* is known as one individual per plant. However, we released 0.5 *N. tenuis* per plant instead due to a lower density of *B. tabaci* in our experimental plots. For the next release, seven adults of *N. tenuis* were released to <*B. tabaci* + *N. tenuis*> and <*B. tabaci* + *N. tenuis* + LED> plots on May 21 and 100 adults of *B. tabaci* were released to all plots on May 30. Lastly, 14 adults of *N. tenuis* were released to <*B. tabaci* + *N. tenuis*> and <*B. tabaci* + *N. tenuis* + LED> plots on June 14.

*Revised sentences*: The optimal release number of *N. tenuis* is known as one individual per plant [30]. However, in the 1^st^ and 2^nd^ predator releases, we used only 0.5 *N. tenuis* per plant due to the low density of *B. tabaci* in our experimental plots. In the last release of *N. tenuis*, one bug per plant was released because the density of mirid bugs at that time was low.

**32. Reviewer comment:** L227: Replace “Between” to “among

**Our response:** Replacement was done. Please check L257 in the revised manuscript.

**33. Reviewer comment:** L230-231: Use the scientific language.

Our response: Revision was done. Please check L260-261 in the revised manuscript.

**34. Reviewer comment:** L231-233: There is no sense in this affirmation. Revise to use the correct statistical analysis! Additionally, You should move this part to statistical analysis section.

**Our response:** Thank you. Before June 14, the insect densities of all plots were almost zero. And, the release of the insect was not finished until that time. Thus we conducted the statistical analysis after that time. We revised the sentence, and it was moved to the data analysis section. Please check L287-293 in revised manuscript.

*Original sentence*: Statistical analysis of weekly density was also performed from June 19 because densities of *B. tabaci* and *N. tenuis* before June 19 were too low to obtain significant results.

*Revised sentence*: In Exp. 4, analysis of the weekly densities of predator and prey were performed from starting on June 19, the first observation date after the last release date (June 14) of *N. tenuis*, because densities of *B. tabaci* and *N. tenuis* before June 19 were too low for analysis.

**35. Reviewer comment:** L239-240: It seems results! Revise!

**Our response:** Thank you. We revised the sentence. Please check L268-269 in the revised manuscript.

*Original sentence*: In experiment 1, the effect of 385 nm wavelength on the density of *N. tenuis* was not significant.

*Revised sentences*: In the Exp. #5, we assessed the effect of the presence or absence of LED light (385 nm) on predation and control of *B. tabaci* by *N. tenuis*.

**35. Reviewer comment:** L242-248: You should provide this information in your table.

**Our response:** We added the information about the released plot in Table 1. And, we deleted the content of the release date and plot in L242-248 of the original manuscript. However, we think that the remaining other part is better. Please check L270-277 in the revised manuscript.

*Original sentences*: All plots (LED or non-LED) received adults of both *B. tabaci* and *N. tenuis*. Thus, each treatment had six replications in three greenhouses. Tomato seedlings (about 300 mm in height) were planted on August 28 and 100 *B. tabaci* adults were released to each plot on Aug 29. Before releasing *B. tabaci*, five to ten *B. tabaci* adults per plant were already present in each plant. Thus, we released 20 *N. tenuis* adults to each plot on September 3. Weekly mean densities of *B. tabaci* and *N. tenuis* were compared between non-LED and LED treatments.

*Revised sentences*: There were two treatments (LED or non-LED light) and all four plots in each of the three greenhouses received adults of both *B. tabaci* and *N. tenuis* (Table 1). Thus, each treatment had six total replications, two in each of the three greenhouses. Tomato seedlings (about 30 cm in height) were planted on August 28. Before our first release of *B. tabaci*, we observed that there were five to ten adult whiteflies per plant already on the tomato plants. Thus, we released 20 *N. tenuis* adults in each plot to achieve the desired predator/prey ratio. Weekly mean densities of *B. tabaci* and *N. tenuis* were compared between non-LED and LED treatments.

**36. Reviewer comment:** Table 3 – The statistical analysis in no response results looks wrong. Are you sure that all is letter a?

**Our response:** Yes, the statistical values of the no response rate in *B. tabaci* are here.

*F*_4, 20_ = 2.27, *P* = 0.0977

And, this graph is distribution of rate made by PROC ANOVA in SAS.

Thus, the non-significant result of no response rate in *B. tabaci* seems to be occurred by high variation of data.

**37. Reviewer comment:** L257-258: It seems discussion.

**Our response:**

The line 257-258 you mentioned is probably lines 357-358 because L257-258 is simply a subheading ‘**Y-tube experiment’** and a statement ‘Results of the Y-tube experiment for *N. tenuis* are presented in Table 2’ .

.

We think you probably intended L357-358.

The sentence of L357-358 is “It might be due to a later colonization of *N. tenuis* caused by an indeterminate reason such as temperature.”. We moved that sentence to our discussion with some revision (L481-483 in the revised manuscript).

*Revised sentence*: This lack of impact on the predator may have been due to the later colonization of *N. tenuis* of our plots due to low temperature or low whitefly density early in the trial.

**38. Reviewer comment:** L279-287: These results could fit well in one table.

**Our response:** Similarly, L279-287 might be L379-387. Thank you for the suggestion. We made Table 5 for those values, and we deleted those in the manuscript. Please check L408, 413 in the revised manuscript.

**39. Reviewer comment:** L411-415: Very confusing! Why it was choose the predator preference?

**Our response:** Thank you for the question. First of all, there was no significant difference between 365 and 385 nm wavelengths in both N. tenuis and B. tabaci. As previously mentioned in this response letter, it is tough to induce just one species' attraction response to certain wavelength light with high efficiency. Thus, we consider the spatial congruence of them, N. tenuis and B. tabaci. And our purpose of this study is to increase the control efficacy of natural enemy by enhancing establishment. Thus, we focused on the response of N. tenuis. Also, we thought that 365 nm wavelength light would be a proper wavelength for this strategy. Please check L82-95 in the revised manuscript. It is probably helpful.

**40. Reviewer comment:** L449-450: Explain better.

**Our response:** Thank you. We revised our sentence. Please check L486-487 in the revised manuscript.

*Original sentence*: One reason could be different environmental conditions.

*Revised sentence*: Differences in environmental conditions between the two greenhouse trials may have occurred.

**41. Reviewer comment:** L460-462: Reference

**Our response:** Thank you. Actually, the reference for this sentence is our study, Fig 3. Thus, we added (Fig 3) at the end of the sentence. Please check L504 in the revised manuscript.

**Reviewer’s comments and our responses (Reviewer #2)**

**1. Reviewer comment:** I read this interesting paper with pleasure. A part from some minor needed corrections (mostly on writing, citations and small details) pointed out in the specific comments below, I have a very major concern on the field assay. In that trial authors did not ensure the isolation of the crop with the environment around, this led major doubts on the obtained results. Moreover, in the first attempt they released a very few predators that did not allow to get reliable results. Finally, a varying number of insect pest was used among the two field trials. Unfortunately, all of this does allow to have a sound experimental setup. Therefore, my best suggestion is to delete (from all relevant sections, included the title) the greenhouse part of this manuscript.

**Our response:** Thank you for the comments. We sincerely appreciate your suggestions. We revised our manuscript by referring to your suggestion. And, we realized that the English of our manuscript has a problem. Thus, our manuscript was professionally re-edited for English by "VanDriesche Scientific Editing"

Our test plots in greenhouse experiments were well isolated from the environment around. We missed the mention about the nylon screen in the window. Thus we added a sentence in our M & M section ("Nylon screening was also used to cover the vents."). Please check L226 in the revised manuscript.

We tried to form an environment as close to reality as possible. We think that you worried about the external inflow of *B. tabaci* in the Exp. #5 (revised version of experiment 2). But, complete blocking of *B. tabaci* inflow to the greenhouse is very hard. As you know, it is almost impossible. And this is a common occurrence in a real farmer's greenhouse. Of course, we can use a denser screen for blocking. However, in this case, ventilation capability would be decreased, potentially harmful to the plant.

**2. Reviewer comment:** L16: I suggest this first sentence: “The zoophytophagous mirid bug *Nesidiocoris tenuis* is a biological control agent of the whitefly *Bemisia tabaci*……”

**Our response:** Thank you for the suggestion. We changed our sentence according to your suggestion. Please check L18-19 in the revised manuscript.

**3. Reviewer comment:** L18: “the” may be added between “with” and “pest”

**Our response:** Thank you. We changed our sentence according to your suggestion. Please check L21 in the revised manuscript.

**4. Reviewer comment:** L24: which wavelength? The same? May be “this wavelength” or “the same wavelength”?

**Our response:** Thank you. We added “this” in front of “wavelength”. Please check L28 in the revised manuscript.

**5. Reviewer comment:** L28-29: I suggest: “in early establishment of this predatory mirid bug,…” “and proper control of the whitefly”

**Our response:** Thank you. We changed the sentence. Please check L33-35 in the revised manuscript.

**6. Reviewer comment:** L33: add “on” before “them”

**Our response:** Thank you. We added “on” before “them”. Please check L39 in the revised manuscript.

**7. Reviewer comment:** L35: add “the” before “tomato”

**Our response:** Thank you. We added “the” before “tomato”. Please check L41 in the revised manuscript.

**8. Reviewer comment:** L38: change “*B. tabaci*” to “this insect pest”. Please revise the whole manuscript for this. Indeed, it is not nice and elegant writing so many times the same word, even though is a species names.

**Our response:** Thank you. Revise done. And also, we revised the whole manuscript for this. Please check L44 in the revised manuscript.

**9. Reviewer comment:** L38: add “strategies” between “control” and “has”

**Our response:** Thank you. We already changed that sentence according to the suggestion of Reviewer 1. Thus, we added the “strategies” after “alternative control”. Please check L45 in the revised manuscript.

**10. Reviewer comment:** L43: add “pest” before “biological control”

**Our response:** Thank you. Add done. Please check L49 in the revised manuscript.

**11. Reviewer comment:** L45: here it could be good if authors cite also two major and recent review articles in which the role of *N. tenuis* on *T. absoluta* predations is emphasized (doi: 10.1146/annurev-ento-031616-034933; doi: 10.1127/entomologia/2018/0749)

**Our response:** Thank you for the suggestion. We cited suggested work in our manuscript. Please check L51, 606, and 610 in the revised manuscript.

**12. Reviewer comment:** L49-52: I suggest authors to include here a statement about the study of Naselli et al. 2017 (https://doi.org/10.1007/s11829-016-9481-5) who evaluated the potential of plant volatiles belonging from alternative host plants as useful tool for managing the mirid behaviour and potentially reduce its feeding activity on cultivated (i.e. tomato) plants.

**Our response:** Thank you for the suggestion. We read the suggested paper. Of course, we can understand the purpose of the study. However, the suggested study focused on olfactory response without field trial and verification of feeding damage decrease to plant. So, this paper was not included. However, we included a suggested statement and reference in our discussion. Please check L427-429, 515-518, and 674 in the revised manuscript.

**13. Reviewer comment:** L54: citations on prey and alternative prey availability need to be included (e.g., DOI 10.1007/s10526-015-9700-5. Doi: 10.1127/entomologia/2019/0824). Moreover, s the role of alternative and banker plants would need to be at least mentioned here as well (e.g., DOI: 10.1127/entomologia/2019/0625; doi: DOI: 10.1080/09670874.2012.659229)

**Our response:** Thank you for the suggestion. We added a sentence with suggested references. Please check L63-70, 600, 639, 642, and 644 in the revised manuscript.

*Added sentence*: Alternative food sources and use of banker plants can be used to help conserve natural enemies.

**14. Reviewer comment:** L56-58: confusing statements. Please rephrase

**Our response:** Thank you. Rephrase done. Please check L68-70 in revised manuscript.

*Original sentence:* To enhance settlement and persistence of natural enemies, escape of natural enemies should be minimized in the absence of food, and their aggregation should be maximized in the target area.

*Revised sentence*: Also, it is helpful to minimize emigration of natural enemies leaving the crop and to maximize natural enemy aggregation where pests are most abundant.

**15. Reviewer comment:** L67: change “establishment of *N. tenuis*” to “establishment of this predatory mirid bug”

**Our response:** Thank you. In the English editing process, the original sentence was corrected. Please check L82-85 in the revised manuscript.

*Original sentence*: If this LED strategy can be applicable to *N. tenuis* in greenhouses, it could enhance the establishment of *N. tenuis* in target areas and help control pests such as *B. tabaci*.

*Revised sentences*: Given that this strategy [44] was successful in open agricultural fields, we hypothesized that the same use of LED light sources should work equally well in greenhouses. Our goal, therefore, was to assess the use of LED lights for enhancement of the impact of *N. tenuis* in greenhouses for control of pest (i.e., *B. tabaci*).

**16. Reviewer comment:** L68: change “help control pests such as *B. tabaci*” to “help pest (i.e., *B. tabaci*) control”

**Our response:** Thank you. In the English editing process, the original sentence was corrected with the suggestion. Please check L82-85 in the revised manuscript.

*Original sentence*: If this LED strategy can be applicable to *N. tenuis* in greenhouses, it could enhance the establishment of *N. tenuis* in target areas and help control pests such as *B. tabaci*.

*Revised sentences*: Given that this strategy [44] was successful in open agricultural fields, we hypothesized that the same use of LED light sources should work equally well in greenhouses. Our goal, therefore, was to assess the use of LED lights for enhancement of the impact of *N. tenuis* in greenhouses for control of pest (i.e., *B. tabaci*).

**17. Reviewer comment:** L68-69: In my opinion the sentence “In addition, ….. agent.” Is repetitive and not necessary here. I suggest deleting it.

**Our response:** Thank you. It was deleted. Please check L85 in the revised manuscript.

**18. Reviewer comment:** L74-75: as currently written seems that “enhancing spatial coherence … could successfully control *B. tabaci*”. I think a verb is needed between “successfully” and “control” (maybe improve, ameliorate, etc). Alternatively sentence modifications should be done to make sense to the statement.

**Our response:** Thank you. In the English editing process, the original sentence was corrected with the suggestion. Please check L90-95 in the revised manuscript.

*Original sentence*: Therefore, the objective of this study was to explore the proper wavelength of LED light to attract both *B. tabaci* and its predator, *N. tenuis*, and determine if enhancing spatial coherence of these two species using LED light could successfully control *B. tabaci* in a tomato greenhouse.

*Revised sentence*: The specific objectives of this study, therefore, were to determine if selected wavelengths of LED light would enhance establishment of *N. tenuis* and thus improve biocontrol of *B. tabaci* in a tomato greenhouse. Additionally, the responses of *B. tabaci* to selected wavelengths of LED light were explored to determine their impact on the spatial congruence of the distributions of natural enemies and the target pest.

**19. Reviewer comment:** L77: change “Methods” to “methods”

**Our response:** Change done. Please check L97 in the revised manuscript.

**20. Reviewer comment:** L78: “Test insects” does not sound good to me. Please provide a better subtitle

**Our response:** We changed to “Insects sources and rearing”. Please check L98 in the revised manuscript.

**21. Reviewer comment:** L82. Spell out the whole genus name after “use.”

**Our response:** Thank you for the suggestion. However, according to your suggestion of #22, we added some words in front of *N. tenuis*. Thus, we did not change. Please check L103-104 in the revised manuscript.

**22. Reviewer comment:** L82: Please specify if the commercial *N. tenuis* specimens were used only to start the laboratory rearing. It is not clear in the current sentence text.

**Our response:** We mentioned the purpose of *N. tenuis* rearing after rearing methods. And, we recognized it could occur confusion to the reader. Thus, we added “For use in the incubator experiments (Exps. #2 and #3),” before “*N. tenuis*” and deleted the sentence “This *N. tenuis* adults were used for incubator experiments.”. Please check L103 and 110 in the revised manuscript.

**23. Reviewer comment:** L89: change “This” to “These” or start the sentence with some words like “Newly obtained *N. tenuis* adults…” or similar.

**Our response:** Thank you. However, we deleted that sentence according to the revision of reviewer comment #22. Please check L110 in the revised manuscript.

**24. Reviewer comment:** L90: see the comment of line 82

**Our response:** Thank you. We spelled out a genus name. Please check L110 in the revised manuscript.

**25. Reviewer comment:** L92: provide mean values ± S.D. or S.E. if possible

**Our response:** Thank you for your suggestion. Unfortunately, we do not have that data. Because the rearing place of *B. tabaci* was not where we managed it.

**26. Reviewer comment:** L100-101: provide specifications about position, size, orientation etc. of the transparent sticky trap inside Y-tube branches

**Our response:** Thank you. We revised and added sentences. Please check L121-124 in the revised manuscript.

*Original sentence*: At each lighting zone, a transparent sticky trap was installed.

*Revised sentences*: At the end of each branch, a transparent sticky trap (40 mm dia) was installed. At the center of each of these transparent sticky traps, a hole (7 x 7 mm^2^; width x length) was drilled to reduce the interference of the trap for the light single.

**27. Reviewer comment:** L102: how these LED light were applied (position, distance, lighted surface)? please specify

**Our response:** We added the sentences about your suggestion. Please check L124-126 in the revised manuscript.

*Added sentence:* The LED lights were installed in cups that inserted tightly into the Y-tube branches’ ends (45 mm ext. dia). The distance between the transparent sticky traps and the LED light source was 10 mm.

**28. Reviewer comment:** L109: specify if 30 specimens were used for each replication (L106) (for a total of 150 specimens per tested wavelength) or in total. It is not completely clear.

**Our response:** Thank you for the suggestion. We changed “test” to “replication”. About 30 *N. tenuis* were used for each replication. Please check L135 in the revised manuscript.

**29. Reviewer comment:** L110-111: 50 *B. tabaci* specimens per tested wavelength in total? had you replications? Please provide more details about this relevant trial aspect

**Our response:** Thank you for the suggestion. We changed “test” to “replication”. About 50 *B. tabaci* were used for each replication. Please check L138 in the revised manuscript.

**30. Reviewer comment:** L117-118: confusing title, please rephrase

**Our response:** We changed the title by dividing two parts. Please check L146 and L204 in the revised manuscript.

*Original title:* Incubator experiment for verification of enhanced establishment rate

*Revised title:*

Exp. #2: No-choice test of effect of light treatment on *N. tenuis* movement and predator efficiency

Exp. #3: Choice test of effect of light treatment on *N. tenuis* movement and predator efficiency

**31. Reviewer comment:** L178: the “Experimental design and sampling method” section is too long and sometimes redundant. Statement such as “Most UV-C from the sun is absorbed into the ozone layer.” could be deleted. General contents should focus on relevant aspects. I suggest rephrasing most of this section by writing concisely and linearly. Sentences of lines 202-206 should come before those of lines 199-202.

**Our response:** Thank you. We deleted that sentence. And moved those sentences. And, we rephrased our manuscript by English re-editing from “VanDriesche Scientific Editing”. Please check L218, 236-241 in the revised manuscript.

**32. Reviewer comment:** L228: you stated “June 19, the last release date of N. tenuis” but the last release was “on June 14”. Please verify and/or correct

**Our response:** Thank you. We revised that part of the sentence. Please check L257-259 in the revised manuscript.

*Original part of sentence:* the control value was calculated from June 19, the last release date of *N. tenuis*,

*Revised part of sentence*: the control value was calculated from June 19, the first observation date after the last release date (June 14) of *N. tenuis*,

**33. Reviewer comment:** L263-265: I suggest deleting percentage values from the statement “Five wavelengths attracted … > 405 nm (41.6%).” because it is repetitive. Indeed, reported results occur in table 2 and some in lines 261-262. (may be better: “385 nm > 365 nm > 445 ………”)

**Our response:** Thank you for the suggestion. We deleted. Please check L302-303 in the revised manuscript.

**34. Reviewer comment:** L268: I suggest adding a specification about the statement “except for wavelength at 445 nm”. As it is currently written, it could seem that 445 nm was the most attractive while it was the least attractive. Please modify or provide specifications

**Our response:** We added “, which showed the lowest attraction rate (37.6%)” after “445 nm”. Please check L307 in the revised manuscript.

**35. Reviewer comment:** L330-332: P values do not confirm that “not significantly different”. Verify and clarify

**Our response:** Actually, that is the results of RM ANOVA, not post hoc test (Tukey’s studentized range test). We presented the results of post hoc test in Fig 3. In Fig 3, there were four kinds of plots, and we just mentioned only three plots in that sentence.

**36. Reviewer comment:** L337-339: this should be moved to the discussion

**Our response:** Thank you for the suggestion. We moved that sentence to our discussion section with some revision. Please check L476-479 in the revised manuscript.

*Original sentence*: These results indicate that *B. tabaci* seems to have a different trend of population dynamics under 385 nm wavelength LED light depending on the presence or absence of natural enemies.

*Revised sentences*: In the first greenhouse trial (Exp. #4), we found that 385 nm wavelength LED lights seems to enhance the establishment rate of *B. tabaci* in absence of predator releases, however, this effect was reversed when both lighting and predators were employed together (Fig 3).

**37. Reviewer comment:** L354-355: “seemed to help the control the activity of *N. tenuis*”. Provide correction

**Our response:** Revision done. We revised that sentence. Please check L387-388 in the revised manuscript.

*Original sentence*: In *N. tenuis* released plots, 385 nm wavelength LED light seemed to help the control the activity of *N. tenuis* for *B. tabaci*.

*Revised sentence*: In the *N. tenuis* release plots, use of 385 nm LED light seemed to enhance predation by *N. tenuis* on *B. tabaci*.

**38. Reviewer comment:** L357-358: these statements should be moved to the discussion

**Our response:** Thank you for the suggestion. We moved that sentence to our discussion section with some revision. Please check L479-483 in the revised manuscript.

*Original sentence*: It might be due to a later colonization of *N. tenuis* caused by an indeterminate reason such as temperature.

*Revised sentences*: LED lighting at a wavelength of 385 nm seemed to increase the control efficacy of the natural enemy (Exp. #4, Fig 5). However, the densities of predator in our first greenhouse trial (Exp. #4) was not affected by LED treatment. This lack of impact on the predator may have been due to the later colonization of *N. tenuis* of our plots due to low temperature or low whitefly density early in the trial.

**39. Reviewer comment:** L390-391: this statement should be moved to the discussion

**Our response:** This statement is already mentioned in our discussion. Thus, we deleted that sentence. Please check L411 in the revised manuscript.

**40. Reviewer comment:** L411-412: spell out the genus after the point “preference. N.”

**Our response:** Thank you. Revised done. Please check L440 in the revised manuscript.

**41. Reviewer comment:** L452-454: confusing sentence. Rephrase

**Our response:** Thank you. We revised our sentence. Please check L494-495 in the revised manuscript.

*Original sentence*: In the plot with 385 nm wavelength LED light after the release of *N. tenuis* and *B. tabaci*, the 385 nm wavelength LED light appeared to enhance the establishment of *N. tenuis*.

*Revised sentence*: In plots with supplemental 385 nm wavelength LED light and the release of both *N. tenuis* and *B. tabaci*, the LED light appeared to enhance the establishment of *N. tenuis*.

**Reviewer’s comments and our responses (Reviewer #3)**

**1. Reviewer comment:** The manuscript “Increase of control efficacy of *Nesidiocoris tenuis* (Hemiptera: Miridae) in the greenhouse by enhancing its establishment using UV-LED light”, study the attraction and effect of Led Light on *Nesidiocoris tenuis* and the herbivore *Bemisia tabaci*, under laboratory and greenhouse conditions. In general, the objective of the work seems interesting to me. However, I have doubts about recommending publishing this paper in PlosOne.

In general, I found some problems with this manuscript in terms of title, the purpose of study, experiments, and results from interpretation. Thus, I really have some concerns about this work, which could be a problem to publish in this journal.
At least for this reviewer, the ms needs a review of the English language. Furthermore, some wordings are challenging to understand ("spatial coherence", “settlement”, "spatial separation", etc…).

**Our response:** Thank you for the comments. We sincerely appreciate your suggestions. We tried to supplement our manuscript and respond sincerely to your comments. According to reviewers’ suggestion, our manuscript was professionally re-edited for English by "VanDriesche Scientific Editing".

**Title**

**2. Reviewer comment:** The title is tough to understand, at least for this reviewer. I suggest to try with something like this: “LED lights enhance the establishment and biological control efficacy of *N. tenuis*”.

**Our response:** Thank you for the suggestion. We changed our title after referring to your opinion. Please check L1-2 in the revised manuscript.

*Original title*: Increase of control efficacy of *Nesidiocoris tenuis* (Hemiptera: Miridae) in the greenhouse by enhancing its establishment using UV-LED light

*Changed title*: UV-LED Lights Enhance the Establishment and Biological Control Efficacy of *Nesidiocoris tenuis* (Reuter) (Hemiptera: Miridae) in the Greenhouse

**Introduction**

**3. Reviewer comment:** Authors introduce both insects well; however, at least for this reviewer, the introduction section should have a better hypothesis of the work and made the objectives clear.

**Our response:** Thank you. We tried to improve our introduction according to reviewers’ suggestion. Please check L86-95 in the revised manuscript.

**4. Reviewer comment:** Line 40-42: This paragraph should move below after to introduce *N. tenuis*.

**Our response:** Thank you. We moved those sentences to the end of the paragraph with revision. Please check L60-62 in the revised manuscript.

*Original sentences*: Generalist natural enemies have alternative food sources besides their target pests. Thus, they have higher adaptability to sustain in the target area than specialist natural enemies.

*Revised sentences*: Because generalist natural enemies can subsist on alternative food sources when the target pest is rare, they can be more adaptable to changing circumstances in the crop than specialist natural enemies.

**5. Reviewer comment:** Line 50-52: There are also alternative food sources as sugars that reduce this damage in tomato plants, authors should be a reference to these works...

**Our response:** Thank you for the suggestion. We found and read the paper (doi.org/10.1111/jen.12151). And, we revised our sentence with reference. Please check L60 and 631 in the revised manuscript.

*Original sentence*: Typical studies include reducing its damage to plants using an endophytic strain *Fusarium solani* and having a proper release density for *N. tenuis.*

*Revised sentence*: Related studies have been conducted to reduce the mirid’s risk to crop plants, including studies of plant resistance to *N. tenuis* mediated by endophytic strain of *Fusarium solani* K, application of sugar as a complementary alternative food, and optimizing the release density for *N. tenuis*.

**6. Reviewer comment:** Line 59: There are also alternative food sources as sugars that improve the establishment of *N. tenuis* in tomato plants; authors should be a reference to these works...

**Our response:** Thank you for the suggestion. We revised our sentence. Please check L70-74 in the revised manuscript.

*Original sentence*: Supplementary food spray using nectar plant and release of natural enemies to the transplant tray a few days before planting can be attempted.

*Revised sentence*: Manipulations tested to improve the spatial association of natural enemies with the pests they attack include the release of the natural enemies onto the transplant trays (when plants are most concentrated) a few days before planting and application of supplementary foods such as plant nectar or sugar to areas with high pest infestations.

**7. Reviewer comment:** Line 66: Authors should make the hypothesis of the study more clear

**Our response:** Thank you. We supplemented that part with the help of the reviewer and English editing company. Please check L82-95 in the revised manuscript.

*Original*: If this LED strategy can be applicable to *N. tenuis* in greenhouses, it could enhance the establishment of *N. tenuis* in target areas and help control pests such as *B. tabaci*. In addition, it can increase the value of *N. tenuis* as a biological control agent. However, we have to consider the reactions of pest species to selected wavelength. If a selected LED light shows asymmetrical response between *N. tenuis* and its prey, it could result in spatial separation between the predator and the prey. Therefore, the objective of this study was to explore the proper wavelength of LED light to attract both *B. tabaci* and its predator, *N. tenuis*, and determine if enhancing spatial coherence of these two species using LED light could successfully control *B. tabaci* in a tomato greenhouse.

*Revision*: Given that this strategy [44] was successful in open agricultural fields, we hypothesized that the same use of LED light sources should work equally well in greenhouses. Our goal, therefore, was to assess the use of LED lights for enhancement of the impact of *N. tenuis* in greenhouses for control of pest (i.e., *B. tabaci*). However, the reaction of key pest species to selected wavelength also have to be considered. If a selected LED light is asymmetrically attractive between *N. tenuis* and its prey, the spatial separation between the predator and the prey, rather than being reduced, might increase. Therefore, we hypothesized that the best strategy would be to select LED light wavelengths that have high attractiveness for both the predator and prey. The specific objectives of this study, therefore, were to determine if selected wavelengths of LED light would enhance establishment of *N. tenuis* and thus improve biocontrol of *B. tabaci* in a tomato greenhouse. Additionally, the responses of *B. tabaci* to selected wavelengths of LED light were explored to determine their impact on the spatial congruence of the distributions of natural enemies and the target pest.

**8. Reviewer comment:** Line 72: Why the authors want to attract *B. tabaci* to tomato plants?? Could this attraction be a detriment for *N. tenuis* biocontrol?

**Our response:** Thank you for the nice question. However, unlike pheromone that can attract specific species, it is tough to induce attraction response to one species using a certain wavelength of light. There were many reports about the positive response for UV or near-UV for pests and natural enemies. Some wavelengths of those ranges were finally chosen for enhancing the establishment of natural enemies [1-3]. In these results, applying a certain wavelength to attract specific species of insects might be hard. Thus, it would be better to consider the spatial congruence of both pests and natural enemies for enhancing the efficacy of biological control. Please check L82-95 in the revised manuscript. It is probably helpful.

In our greenhouse experiment, we closed side windows of greenhouses when LED lights were turned on, and the windows were opened after sunset to reduce the external inflow of *B. tabaci* by lighting.

References

1. Johansen NS, Vänninen I, Pinto DM, Nissinen AI, Shipp L. In the light of new greenhouse technologies: 2. Direct effects of artificial lighting on arthropods and integrated pest management in greenhouse crops. Ann Appl Biol. 2011; 159(1): 1-27.
2. Ogino T, Uehara T, Muraji M, Yamaguchi T, Ichihashi T, Suzuki T, et al. Violet LED light enhances the recruitment of a thrip predator in open fields. Sci Rep. 2016; 6: 32302.
3. Uehara T, Ogino T, Nakano A, Tezuka T, Yamaguchi T, Kainoh Y, et al. Violet light is the most effective wavelength for recruiting the predatory bug *Nesidiocoris tenuis*. BioControl. 2019; 64(2): 139-147.

**M&M**
**9. Reviewer comment:** Line 81: “25ºC” needs (± SE). Here and throughout the manuscript.

**Our response:** Before we used it, the incubator’s temperature was set at 25 ºC for a long time. And, in our opinion, the storage time was not too long. Thus, we didn’t check the variation of temperature for this method.

**10. Reviewer comment:** Line 82: “at 25ºC for ca. 1h before use”, at least for this reviewer, this is a little bit misleading if these *N. tenuis* are for rearing.

**Our response:** Thank you for the comment. Actually, that condition was set for storage of *N. tenuis* before Y-tube test. We changed “ca.” to “about”. And, we added, “For use in the incubator experiments (Exps. #2 and #3),” to the beginning of the next sentence. Please check L102-103 in the revised manuscript.

**11. Reviewer comment:** Line 88: Why the authors use eggs of *C. cautella* to evaluate the predation rate instead of other eggs source (e.g. *Tuta absoluta*, *Ephestia kuehniella*)?

**Our response:** In our situation, using eggs of *C. cautella* was the best option. Because we can easily buy the eggs when we want from Osang Kinsect System. And, *Nesidiocoris tenuis* is a generalist predator, and it can use most of Pyralid moth eggs as prey. Moreover, *Tuta absoluta* is not live in Korea.

**12. Reviewer comment:** Line 89: Which *N. tenuis*? How old are *N. tenuis* used in ALL the experiments? Are males or females?

**Our response:** We deleted that sentence according to the suggestion of Reviewer 2. Instead, we added, “For use in the incubator experiments (Exps. #2 and #3),” at the beginning of the rearing part. For the experiments, we collected *N. tenuis* by random. Please check L103 and 110 in the revised manuscript.

**13. Reviewer comment:** Line 96: Although the Y-tube results seem that the response of *B. tabaci* and *N. tenuis* are reliable enough. I think that the methodology of this experiment needs to be more explicit. How the authors separated the “lighting zone”? Is there any work that used this methodology for light attraction?

**Our response:** In our Y-tube, there were three branches. It was made by 3D printing of plastic, and the outer cover of the Y-tube was painted in black. Chiel et al. (2006) used a similar method. We newly cited Chiel et al. (2006). And we revised and added a sentence for this. Please check L118-120, 130-132 and 667 in the revised manuscript.

Reference

Chiel E, Messika Y, Steinberg S, Antignus Y. The effect of UV-absorbing plastic sheet on the attraction and host location ability of three parasitoids: *Aphidius colemani*, *Diglyphus isaea* and *Eretmocerus mundus*. BioControl. 2006; 51: 65–78.

*Original sentence*: During the test, each branch of the Y-tube was capped to block penetration of light from the outside.

*Revised sentences*: During a test, each branch of the Y-tube was capped to prevent penetration of light from the outside into the test arena. The body of the Y-tube made of plastic (3D printing) that did not permit light to pass.

**14. Reviewer comment:** Line 100: “branches” means “arms”?

**Our response:** Yes.

**15. Reviewer comment:** Line 108: “About 30 *N. tenuis*”?. The authors need to be more concise.

**Our response:** For every replication of our Y-tube test, we collected the insects using an electric aspirator by counting the number of insects. Some insects were escaped in the process of input to Y-tube, and sometimes, insects were collected more than that we counted. It was tough to count in the bottle of aspirator because it was always moving. Of course, we can make them knock out in a moment by freezing. However, we thought that it would be some stress on them. Moreover, we just counted again after the experiment. So, there was some variance in the insect number. That is why we used “about” or “approximately” in lines 108 and 110 of the original manuscript.

**16. Reviewer comment:** Line 110: “Approximately 50 *B. tabaci*”?. The authors need to be more concise.

**Our response:** Please check our answer for reviewer comment #15.

**17. Reviewer comment:** Line 117: this section evaluates the predation rate. It is okay. However, how the authors evaluate the establishment?

**Our response:** As we mentioned in the last paragraph of that section, the incubator was not sealed. There were many holes for ventilation and environmental control. Thus, the mirid bugs can escape using that hole. In our results, 385 nm wavelength treatment can make more *N. tenuis* stay in the incubator. Thus, we assume that 385 nm wavelength LED light can increase the establishment of *N. tenuis* in a certain area. However, we partly agreed about your comments. Thus, we gave some revision for that part by deleting “establishment”. Please check L150-155 in the revised manuscript.

*Original sentence*: predation amount and establishment rate of *N. tenuis* were compared among different light conditions in the incubator.

*Revised sentences*: In this experiment, we measured the rate of predation by the released mirids on eggs of *C. cautella* as a “predation index”*.* The incubator was not a fully enclosed space. Rather, it had some holes in the inner side wall for environmental controls, and there was a vessel of water behind one wall. Some *N. tenuis* left the experimental arena and drowned in this water. Thus, we also counted the remaining number of mirids in an incubation chamber at the end of each assay.

**18. Reviewer comment:** Line 152: Which is the second experiment?

**Our response:** We divided that section. Exp. #3 is the second experiment. Please check L146 and 204 in the revised manuscript.

**19. Reviewer comment:** Line 198: *N. tenuis* population includes nymphs and adults? Also, for the predation experiment?

**Our response:** In the greenhouse test, *N. tenuis* population includes nymphs and adults. However, in the incubator test, we used the only adult. Because we want to check how many *N. tenuis* can escape in an incubator. The adults have wings, so we thought that adult *N. tenuis* is more suitable for our purpose.

**20. Reviewer comment:** Line 199: all the tomato plant leaves were counted??

**Our response:** Yes, we checked all leaves, stems, and trunks.

**21. Reviewer comment:** Line 203-204: “7 to 20 *N. tenuis* were release”? The number of *N. tenuis* released is essential for results interpretation.

**Our response:** We represented all the release number and date in Table 1. Please check L246 in the revised manuscript.

**22. Reviewer comment:** Line 204-205: same for the number of *B. tabaci*

**Our response:** We represented all release the number and date in Table 1. Please check L246 in the revised manuscript.

**23. Reviewer comment:** Line 221: Calvo et al. 2012 showed that 0.25-0.5 *N. tenuis* per plant was a good release range.

**Our response:** Yes, right. However, that release range is for pre-plant release strategy. Actually, we did not apply that strategy in our experiments.

**Results**

**24. Reviewer comment:** Line 258: Removed this sentence

**Our response:** Thank you for the suggestion. We removed it. Please check L297 in the revised manuscript.

**25. Reviewer comment:** Table 2: It is known that results from Y-tube should be shown in vertical bars figures. For this reason, at least for this reviewer, results from the Y-tube will be better represented by figures (e.g. left side responders to different LED light treatments, right side responders to control treatment, and outside the figure but next to it, the number of individuals who did not respond should be included).

**Our response:** Thank you for the suggestion. We think you might want to revise Table 2 to the figure (extracted from Naselli et al. 2016) below.

In the results of Naselli et al. (2016), almost all insects could make their choice. Thus, they only compared two treatments. However, in our results, the treatments of different wavelengths significantly affected to no response rate. Thus, we think that no response is also an important factor. And, we think representing in the Table is better for comparing among the different wavelength treatments.

Reference

Naselli M, Zappala L, Gugliuzzo A, Garzia G T, Biondi A, Rapisarda C, Cincotta F, Condurso C, Verzera A, Siscaro G. Olfactory response of the zoophytophagous mirid *Nesidiocoris tenuis* to tomato and alternative host plants. Arthropod-Plant Interactions, 2016; 11(2): 121-131.

**Greenhouse experiments**

**26. Reviewer comment:** Why did the authors not use a GLMM to analyze the greenhouse data?? This model can include factors as the date and interaction date*treatment…

**Our response:** Thank you for the comments. For the Generalized linear mixed model (GLMM) in SAS, PROC MIXED should be used. And there are two options for RM ANOVA in SAS, PROC GLM and MIXED. They have pros and cons.

Firstly, we analyzed our greenhouse data by using PROC GLM. And we analyzed it again with PROC MIXED. However, the statistical values (i.e., F-value, df, P-value) were the same. So we just wrote the PROC GLM that we analyzed first in our manuscript. Moreover, PROC GLM also include factors as the date and interaction date*treatment, and we already mentioned the results of date and date*treatment in our results section. Please check L352-355, 372-375, and 402-406 in the revised manuscript.

**27. Reviewer comment:** Line 354: “In *N. tenuis* released plots, 385 nm wavelength LED light seemed to help the control the activity of *N. tenuis* for *B. tabaci*”. However, when you showed the figure, there are no differences between treatments in *B. tabaci* populations…It seems that LED lights did not affect *B. tabaci* control by *N. tenuis*.

**Our response:** Thank you for the comments. Actually, in our Exp. #4 (revised version of greenhouse exp. 1), we can’t find a significant difference in *N. tenuis* density. Thus, we suggested the control value (Fig 5). We think it can be supplementary data for Exp. #4. And we discussed this in our discussion section (L476-489 in the revised manuscript). We thought that this result was caused by two reasons: 1. The Low density of *B. tabaci* and 2. Environmental condition. The initial density of *B. tabaci* in Exp. #4 was very low. Thus *N. tenuis* cannot make their population increase. Also, the early temperature of Exp. #4 was lower than Exp. #5. Low temperature affected to population development of both *B. tabaci* and *N. tenuis*. Thus, we released *N. tenuis* and *B. tabaci* three times in Exp. #4. From June mid, both insect densities were increased in all plots. We could get significant results in the density of *B. tabaci*. However, it was too late to get a significant result for *N. tenuis*. Thus, we concentrated on *N. tenuis* density and its control efficacy in Exp. #5. That is why we organized different plots between Exp. #4 and #5.

**28. Reviewer comment:** Line 368: At least for this reviewer, it is hard to understand how the percentage of control value of *B. tabaci* + *N. tenuis* + LED are higher in all the sample dates if the *B. tabaci* levels are not significantly different.

**Our response:** Thank you for the question. When we calculated the control value, we used *B. tabaci* only plot as a control, and another two plots (*B. tabaci* + *N. tenuis* / *B. tabaci* + *N. tenuis* + LED) were treatments. Mean densities were used for control value without regarding statistical significance. Thus, we can find some differences in our control value. We suggested control value as a supported data for *N. tenuis* and necessity of Exp. #5. Because we cannot get a significant result of *N. tenuis* in greenhouse Exp. #4. We explained the reason in the answer of the above question and our discussion (L476-489 in the revised manuscript).

**29. Reviewer comment:** Line 371: The experiment 2 showed that LED lights' effect on *B. tabaci* populations reductions correlated with *N. tenuis* populations increases. The authors should justify better the results of the first experiment throughout the discussion.

**Our response:** Thank you for the suggestion. Please check L476-489 in the revised manuscript.

*Added sentences*: In the first greenhouse trial (Exp. #4), we found that 385 nm wavelength LED lights seems to enhance the establishment rate of *B. tabaci* in absence of predator releases, however, this effect was reversed when both lighting and predators were employed together (Fig 3). LED lighting at a wavelength of 385 nm seemed to increase the control efficacy of the natural enemy (Exp. #4, Fig 5). However, the densities of predator in our first greenhouse trial (Exp. #4) was not affected by LED treatment. This lack of impact on the predator may have been due to the later colonization of *N. tenuis* of our plots due to low temperature or low whitefly density early in the trial.

**Discussion**

**30. Reviewer comment:** In my consideration, the discussion should be rewritten again. Authors need to be more consistent with their results and then discuss better with other works. There are a large number of sentences that need references.

**Our response:** Thank you for the comments. We supplemented our discussion with re-editing in English. And we added some references. Please check L427-430, 437-438, 451, 468, 476-489, 496, 497, 502, 504, and 518 in the revised manuscript.

**31. Reviewer comment:** Line 421 -422 and Line 433-434 are almost the same sentences. In my opinion, I removed the first one because this sentence here did not match the flow of the discussion.

**Our response:** Thank you for the suggestion. However, supplementary light is not always used by combining red and blue light. We intend in the first sentence (Line 421-422) to use each wavelength separately, and we think that deleting the first sentence will not support the following sentences. The best option we have in mind is to change the latter part of the second sentence (Line 433-434 in the original manuscript). Please check L458-459 in the revised manuscript.

*Original sentence*: Supplementary light for promoting plant growth is often used by combining red and blue light

*Revised sentence*: Supplementary light for promoting plant growth often based on a combination of different wavelengths of light.

**32. Reviewer comment:** Line 439 and line 443: “supported results of the greenhouse experiment” (experiment 1 or 2?)

**Our response:** Thank you. As a result, that is proper content for Exp. #5 (revised version of experiment 2), but we intended and wrote both Exp. #4 and #5 because we obtained similar results from the whitefly in Exp. #4 (revised version of experiment 1).

**33. Reviewer comment:** Line 450: Figure 8. From where are these temperature and humidity data? Which greenhouse? If authors represent each day's abiotic conditions, they should put the temperature, and RH mean and SE.

**Our response:** We installed the data logger in each greenhouse. Thus, we added “of each greenhouse” in M&M. Our data is mean data between observation date. And we revised our figure. Please check L243, 491 in the revised manuscript and newly updated Fig 8.

**34. Reviewer comment:** Line 468-469: Experiment 1 or 2?

**Our response:** It means the whole of our study, not just for the greenhouse experiment.
