# Supplementary material for: UV-LED lights enhance the establishment and biological control efficacy of Nesidiocoris tenuis (Reuter) (Hemiptera: Miridae)
Source: PLoS One. 2021 Jan 7;16(1):e0245165. doi: 10.1371/journal.pone.0245165 (PMC7790406; doi:10.1371/journal.pone.0245165)
Supplement: Attachment — Submitted filename: Response to Reviewers.docx [file pone.0245165.s003.docx]

**Dear Editor**

Thank you for giving us another chance and the helpful comments on our manuscript. We have faithfully revised our manuscript by referring to the reviewer's opinion. After careful consideration, we decided to delete the part of the greenhouse experiment in our manuscript. Along the way, we revised the title to "UV-LED Lights Enhance the Establishment and Biological Control Efficacy of *Nesidiocoris tenuis* (Reuter) (Hemiptera: Miridae)". And, we gave the new number to our figures and table. Moreover, according to the suggestion of Reviewer 3, we changed tables (about the Y-tube test) to figures.

Revised parts for removing the greenhouse experiments, reviewer 2, and 3 were marked in red, green, and orange color in the text, respectively.

**Revised part by deleting the greenhouse experiments**

**1. Title:** revised**.**

*Previous title*: UV-LED Lights Enhance the Establishment and Biological Control Efficacy of *Nesidiocoris tenuis* (Reuter) (Hemiptera: Miridae) in the Greenhouse

*Revised title*: UV-LED Lights Enhance the Establishment and Biological Control Efficacy of *Nesidiocoris tenuis* (Reuter) (Hemiptera: Miridae)

**Abstract**

**2. L23 in the revised manuscript:** “in laboratory condition” added after “establishment of *N. tenuis*”

**3. L28 in the revised manuscript:** deleted “and lights of ~ in greenhouses” after “the attraction of *N. tenuis* to 385 nm wavelength”

**4. L28–31 in the revised manuscript:** revised.

*Original sentence*: When LED light at a wavelength of 385 nm was used in a greenhouse for 6 hours starting at sunset every night, it significantly enhanced the establishment rate of *N. tenuis* and the level of control of *B. tabaci* achieved in comparison to treatment without LED light.

*Revised sentence*: When LED light at a wavelength of 385 nm was used in a growth chamber for 6 hours out of 24 hours, it significantly increased the remaining number of *N. tenuis* in growth chamber and level of predation compared to treatment with white LED light or without LED light.

**5. L31-34 in the revised manuscript:** revised.

*Original sentence*: In conclusion, UV-LED light at a wavelength of 385 nm attracts both *B. tabaci* and *N. tenuis,* resulting in early establishment of this predatory mirid bug, better spatial congruence of both species, and better control of the whitefly*.*

*Revised sentences*: In conclusion, UV-LED light at a wavelength of 385 nm attracts both *B. tabaci* and *N. tenuis*. Thus, it would be used for enhancing early establishment of this mirid bug, better spatial congruence of both mirid bug and whitefly, and better control of the whitefly*.*

**Introduction**

**6. L82-84 in the revised manuscript:** revised.

*Original sentence*: Our goal, therefore, was to assess the use of LED lights for enhancement of the impact of *N. tenuis* in greenhouses for control of pest (i.e., *B. tabaci*).

*Revised sentence*: Our goal, therefore, was to evaluate the use of LED lights for enhancement of the impact of *N. tenuis* in the laboratory condition for pest (i.e., *B. tabaci*) control.

**7. L91 in the revised manuscript:** deleted “and thus improve biocontrol of *B. tabaci* in a tomato greenhouse” after “would enhance establishment of *N. tenuis*”

**Materials and methods**

**8. L148-151 in the revised manuscript:** moved from the greenhouse experiment section.

**9. L215, 224 in the revised manuscript:** the greenhouse part deleted (L214-277 and 287-293 in the original manuscript were deleted).

**Results**

**10. L238-239 in the revised manuscript:** revised.

*Original sentence*: Thus, the 385 nm wavelength was selected for the greenhouse experiment.

*Revised sentence*: Thus, the 385 nm wavelength was selected for the incubator assays.

**11. L281 in the revised manuscript:** the greenhouse part deleted (L350-424 in the original manuscript were deleted).

**Discussion**

**12. L299 in the revised manuscript:** added “and accomplishing their spatial congruence” after “for further test”.

**13. L322 in the revised manuscript:** deleted “and this finding supported the results of our greenhouse experiments” after “incubator assays.”.

**14. L327-328 in the revised manuscript:** revised.

*Original sentence*: These results also support the results of our greenhouse experiments.

*Revised sentence*: Thus, if this LED light is applied in the greenhouse, it may increase the retention time of *N. tenuis* resulting in enhanced establishment of this predator.

**15. L332-333 in the revised manuscript:** Related paragraph and figure legend about the greenhouse experiments were deleted (L476-497 in the original manuscript were deleted).

**16. L337-339 in the revised manuscript:** revised.

*Original sentences*: When the abundance of natural enemies is low due to the use of pesticides or a naturally low number of natural enemies in the crop, lighting condition may accelerate population increase of the target pests (Fig 3). In this study, we closed the greenhouse’s vents during the supplemental lighting period to reduce the attraction of whiteflies into the greenhouse.

*Revised sentences*: Thus, the greenhouse's vents should be closed during the supplemental lighting period to reduce the attraction of whiteflies into the greenhouse, and regular monitoring of both natural enemy and pest densities would be needed.

**17. L342-345 in the revised manuscript:** revised.

*Original sentences*: Results of this study clearly indicate that 385 nm wavelength LED light can improve the efficacy of *N. tenuis* in greenhouses for controlling *B. tabaci*. The 385 nm wavelength LED light appeared to attract *N. tenuis* and enhance early population build-up of *N. tenuis*, resulting in better control of *B. tabaci*.

*Revised sentences*: Results of this study clearly indicate that 385 nm wavelength LED light can improve the efficacy of *N. tenuis* in released area. Also, the 385 nm wavelength LED light could help achieve the spatial congruence of both *N. tenuis* and *B. tabaci* in the target area because this LED light can attract both species.

**18. L353-355 in the revised manuscript:** revised.

*Original sentence*: Further studies are needed to better understand how best to use LED lights, such as optimal lighting time and wattage of lights and assessments of the applicability of such light supplementation in other greenhouse crops.

*Revised sentence*: Further studies are needed to better understand how best to use LED lights, such as optimal lighting time and wattage of lights and assessments of the applicability of such light supplementation in the greenhouse.

**Reviewer’s comments and our responses (Reviewer #2)**

**1. Reviewer comment:** Authors re-submitted a new version of the manuscript where they ameliorated some minor concerns. The current version appears properly written and referenced, even if some changes are still necessary (see specific comments). However, I renew my strong doubts about the methods employed for conducting the greenhouse trials (occurrence of the pest before starting trials, varying number of insect pest released in different times, release of very few predators) and consequently I confirm my first recommendation to delete (from all relevant sections, included the title) the greenhouse part of this manuscript. Indeed, in my opinion, all the above reasons do not allow to have a sound experimental setup.

**Our response:** We deleted the part of the greenhouse experiment in our manuscript. Please check the part "Revised part by deleting the greenhouse experiments" before this. And, we revised our manuscript according to your specific comments.

**2. Reviewer comment:** L21: add “its” before “co-occurrence”
**Our response:** Yes. Please check L21 in the revised manuscript.

**3. Reviewer comment:** L21-22: add “programs” before “in greenhouses”
**Our response:** Yes. Please check L22 in the revised manuscript.

**4. Reviewer comment:** L24: change “crop” to “crops”

**Our response:** Yes. Please check L24 in the revised manuscript.

**5. Reviewer comment:** L67: I suggest to delete “and use” and to say directly “Alternative food sources and banker plants can be used…”

**Our response:** Yes. Please check L66 in the revised manuscript.

**6. Reviewer comment:** L72: add “the” before “application”

**Our response:** Yes. Please check L71 in the revised manuscript.

**7. Reviewer comment:** L85: I suggest to use “for pest (i.e., *B. tabaci*) control”

**Our response:** Yes. Please check L84 in the revised manuscript.

**8. Reviewer comment:** L122 and 125: the use of the abbreviation in “40 mm “dia”” and “45 mm “ext. dia”” does not sound good to me, please check the journal guidelines and make changes if necessary

**Our response:** We revised “dia” to “diameter” and “ext. dia” to “external diameter”. Please check L120 and 123 in the revised manuscript.

**9. Reviewer comment:** L131-132: a verb seems to be missing in this sentence, please correct it
**Our response:** We added the verb “was”. Please check L129 in the revised manuscript.

**10. Reviewer comment:** L218: may be “considered” despite of “consider”?

**Our response:** That line was deleted by removing the greenhouse part in the manuscript.

**11. Reviewer comment:** L220: may be “consider” and “tested” despite of “considered” and “test”?

**Our response:** That line was deleted by removing the greenhouse part in the manuscript.

**12. Reviewer comment:** L227: change contained” to “consisted of”

**Our response:** That line was deleted by removing the greenhouse part in the manuscript.

**13. Reviewer comment:** L246 - Table 1: Were the releases of “*B. tabaci* + *N. tenuis* +LED” the same of “*B. tabaci* + *N. tenuis*”? Please, make the Table more intelligible and clarify/specify the aforementioned doubt

**Our response:** That part was deleted by removing the greenhouse part in the manuscript.

**14. Reviewer comment:** L276: correct “desired”

**Our response:** That was deleted by removing the greenhouse part in the manuscript.

**15. Reviewer comment:** L287: change “Mean” to “mean”

**Our response:** That was deleted by removing the greenhouse part in the manuscript.

**Reviewer’s comments and our responses (Reviewer #3)**

**1. Reviewer comment:** Although the authors have improved the paper compared to the first submission, at least for me, they have not made enough adjustments to have the manuscript ready for acceptance.

My primary concern is the part of the greenhouse experiments. At least for this reviewer, the methodology of greenhouse experiments is misleading and is not strong enough to be published in this journal (i.e., the numbers of the whiteflies and N. tenuis used in the experiments). I read the reviewer 2 comments, and this reviewer recommended removing the greenhouse experiments (which I partly agree with), however authors did not remove it. At least for this reviewer, authors should do the experiment again under greenhouse conditions or try to remove it from the study; however, this reviewer is not sure if the study will be too short.

Furthermore, the answer to points 15 and 16 (of the reviewer 3) made by authors is questionable concerning this topic.
15. Reviewer comment: Line 108: “About 30 N. tenuis”?. The authors need to be
more concise.
16. Reviewer comment: Line 110: “Approximately 50 B. tabaci”?. The authors need to be more concise.
Our response: For every replication of our Y-tube test, we collected the insects using
an electric aspirator by counting the number of insects. Some insects were escaped
in the process of input to Y-tube, and sometimes, insects were collected more than
that we counted. It was tough to count in the bottle of aspirator because it was always
moving. Of course, we can make them knock out in a moment by freezing. However,
we thought that it would be some stress on them. Moreover, we just counted again
after the experiment. So, there was some variance in the insect number. That is why
we used “about” or “approximately” in lines 108 and 110 of the original manuscript.

There are many ways to know the total number of insects used in the experiments. At least for this reviewer, the numbers of insects used for the experiments are very important and help make the study more concise for a potential publication in a scientific journal.

However, I consider that I mistake by correcting when I suggest representing the Y-tube results (Table 2 and 3) in a figure with vertical bars. I want to say horizontal bars (similarly to Naselli's figure that the authors used to answer my suggestion), with the non-responders individuals between brackets in the corresponding bars.

**Our response:** Thank you for the comments. We sincerely appreciate your suggestions. After careful consideration, we decided to delete the part of the greenhouse experiment in our manuscript. Moreover, we tried not to diminish the manuscript much. Please check the part "Revised part by deleting the greenhouse experiments" before the response to Reviewer #2.

Following your suggestion, we changed our tables for the Y-tube test to figures by referring Naselli's figure. Please check our new Figs 2 and 3.

There was a little misunderstanding by us. We exactly know the total number of insects used in our Y-tube test. There were many replications for each insect (*N. tenuis* - 50 times replicated, 5 replications per each wavelength x 10 test wavelengths; *B. tabaci* - 25 times replicated; 5 replications per each wavelength x 5 test wavelengths). Thus, we thought that it was hard to express all initial numbers respectively. Now, we understood your intention. So, we expressed the initial number range for each wavelength treatment instead of all numbers in our new Figs 2 and 3. Please check our new Figs 2 and 3 and L245 and 251 in the revised manuscript.

**2. Reviewer comment:** Change “Exp. #1” to “Experiment 1” throughout all the manuscript, experiments (2, 3, and 4), including the subheadings.

**Our response:** Yes. Please check L97, 101, 115, 144, 160, 205, 207, 209, 210, 217, 220, 226, 242, 248, 253, 255, 267, 271, and 278 in the revised manuscript.

**3. Reviewer comment:** Change “branches” by “arms” about the y-tube.

**Our response:** Yes. Please check L118-120, 123, 126, and 128 in the revised manuscript.

**4. Reviewer comment:** Why are the ±SE of the abiotic conditions (Temperature and relative humidity) too high?

**Our response:** That figure was deleted by removing the greenhouse part in the manuscript.
